# Prostate Intrafraction Translation Margins for Real-Time Monitoring and Correction Strategies

**DOI:** 10.1155/2012/130579

**Published:** 2011-07-13

**Authors:** Dale W. Litzenberg, James M. Balter, Scott W. Hadley, Daniel A. Hamstra, Twyla R. Willoughby, Patrick A. Kupelian, Toufik Djemil, Arul Mahadevan, Shirish Jani, Geoffrey Weinstein, Timothy Solberg, Charles Enke, Lisa Levine, Howard M. Sandler

**Affiliations:** ^1^Department of Radiation Oncology, University of Michigan Health System, Ann Arbor, MI 48109-5010, USA; ^2^M. D. Anderson Cancer Center Orlando, Orlando, FL 32806, USA; ^3^UCLA, Los Angeles, CA 90095, USA; ^4^Cleveland Clinic Foundation, Cleveland, OH 44195, USA; ^5^Sharp Health Care Hospital, San Diego, CA 92123, USA; ^6^University of Texas Southwestern Medical Center, Dallas, TX 75390, USA; ^7^Nebraska Medical Center, Omaha, NE 68198, USA; ^8^Calypso Medical Technologies Inc., Seattle, WA 98121, USA; ^9^Cedars-Sinai, Los Angeles, CA 90048, USA

## Abstract

The purpose of this work is to determine appropriate radiation therapy beam margins to account for intrafraction prostate translations for use with real-time electromagnetic position monitoring and correction strategies. Motion was measured continuously in 35 patients over 1157 fractions at 5 institutions. This data was studied using van Herk's formula of (*αΣ* + *γσ*') for situations ranging from no electromagnetic guidance to automated real-time corrections. Without electromagnetic guidance, margins of over 10 mm are necessary to ensure 95% dosimetric coverage while automated electromagnetic guidance allows the margins necessary for intrafraction translations to be reduced to submillimeter levels. Factors such as prostate deformation and rotation, which are not included in this analysis, will become the dominant concerns as margins are reduced. Continuous electromagnetic monitoring and automated correction have the potential to reduce prostate margins to 2-3 mm, while ensuring that a higher percentage of patients (99% versus 90%) receive a greater percentage (99% versus 95%) of the prescription dose.

## 1. Introduction

The goal of conformal radiation therapy is to shape the dose distribution to the prescribed target volume as closely as possible without sacrificing target coverage. This technique results in the sparing of neighboring healthy tissues and often leads to fewer complications and higher quality of life. It may also allow higher doses to target volumes that are limited by toxicity of normal tissues, potentially resulting in better local tumor control. In the last five years, real-time electromagnetic tracking of the prostate has become commercially available and has been adopted as the preferred localization technique in many clinics where it is available. The technology makes it possible to essentially eliminate interfraction variations, greatly reduces systematic uncertainties, and allows intra-fraction target volume motion to be monitored continuously throughout treatment so that corrective adaptive action may be taken. 

A description of the Calypso System has been previously reported [1, 2]. Briefly, the system consists of a tracking station (placed in the control room) to display real-time deviations for the target volume from isocenter. Ceiling-mounted infrared cameras localize an electromagnetic array which is placed over the patient before and during treatment. The array excites and localizes transponders which are implanted transrectally into the prostate. The transponders are 8 mm long by 1.85 mm in diameter and are implanted transrectally with a 14 gauge needle using similar procedures for obtaining prostate biopsies. Transponders were placed at the apex and right- and left-base under ultrasound guidance. An isocenter was chosen relative to the geometric center of the transponders on CT, and the (supine) patients were positioned to this isocenter using the electromagnetic system. The relative stability of the transponders within the prostate has been previously reported to have a standard deviation of 0.9 to 1.2 mm about their expected separations over the course of therapy [3]. The measurement uncertainties associated with this device are <0.54 mm in all directions for transponders at the far boundary (27 cm from the array), of the 14 cm × 14 cm × 27 cm active tracking volume, and for transponder velocities up to 3.0 cm/s [1]. 

Using the transponders as surrogates for prostate motion, the distribution of deviations from isocenter has been shown to vary widely from patient to patient and from fraction to fraction. For 20 patients, deviations >3 mm occurred 13.6% of the time on average, though individual patients exhibited deviations >3 mm for as much as 36.2% of the time over the course of their therapy. While some patients are extremely stable on any given fraction, at other times or in other patients, deviations >3 mm occurred 98.7% of the time [4]. Based on limited initial data, margins were estimated from a data set where prostate motion was tracked continuously in 11 patients for 8–10 minutes each [5]. (To simulate a treatment fraction, each of the patients were setup in a treatment room in a clinical supine treatment position, though no treatment was delivered.) Margin estimates varied depending on the amount of corrective action taken, ranging from approximately 7 mm for single daily pretreatment positioning, to 1.5 mm when positioning was corrected to isocenter when a 3 mm action threshold was exceeded.

A multi-institutional clinical study has since been conducted [6] in which electromagnetic guidance was used daily on over 40 patients who were enrolled in IRB-approved protocols. Continuous tracking during therapy was conducted in 35 patients. In the study presented here, the PTV margins required to account for intra-fraction target volume motion, for a variety of real-time correction strategies, are studied using data from real-time (~10 Hz) electromagnetic tracking with the Calypso System (Calypso Medical, Seattle, Wash, USA), based on this much larger multi-institutional clinical dataset. At the time this study was conducted, the system was an investigational device but has since received FDA 510(k) clearance and is now commercially available.

## 2. Materials and Methods

Under IRB-approved protocols, 35 patients at 5 institutions were continuously tracked during treatment to study intra-fraction prostate motion using the Calypso System in which 3 Beacon electromagnetic transponders are implanted into the prostate. The prostate was initially aligned using skin marks and lasers. The Calypso System was then used to localize the prostate based on predetermined transponder positions relative to isocenter on the treatment planning CT. The deviations of the prostate from isocenter were measured continuously (10 Hz) during 1157 fractions.

### 2.1. Analysis

This data was studied, using the method of van Herk [7, 8] to determine appropriate clinical target volume (CTV) to planning target volume (PTV) margins under various conditions. By this methodology, CTV to PTV margins, **m**, are given by

(1) $$m=\alpha \text{Σ}+\gamma \sigma^{\prime },$$

where *α* and *γ* specify the confidence level in determining the margins due to systematic and random errors, and $\text{Σ}=\sqrt{{\text{Σ}}_{s,\;\text{Inter}}^{2}+{\text{Σ}}_{\text{Intra}}^{2}}$ and $\sigma^{\prime }=\sqrt{\sigma_{s,\;\text{Inter}}^{2}+\sigma_{\text{Intra}}^{2}}$, are respectively, the standard deviation of all appropriate preparation (systematic) and treatment (random) errors for a population of patients, added in quadrature. (In this methodology, preparation errors are defined as those that lead to a displacement of the dose distribution with respect to the CTV while treatment errors lead to a blurring of the dose distribution.) The subscripts, “*s*,” “Inter,” and “Intra,” denote setup, interfraction motion, and intra-fraction motion, respectively. The systematic errors associated with initial patient setup and inter-fraction variation of internal anatomy are given by Σ_*s*, Inter_ while Σ_Intra_ is the systematic error associated with average daily changes during treatment. Likewise, the random errors associated with initial patient setup and inter-fraction variation of internal anatomy are given by  *σ*_*s*, Inter_ while *σ*_Intra_ is the random error associated with average daily changes during treatment. 

Commonly used values of *α* = 2.5 and *γ* = 0.7 were used to estimate margins, **m**_90,95_, where 90% of patients would receive a minimum dose of 95% of the prescription dose. In addition, values of *α* = 3.36 and *γ* = 0.95 were also used to estimate margins, **m**_99,99_, where 99% of patients would receive a minimum dose of 99% of the prescription dose. Margins were estimated for situations of skin-based positioning (a) with and (b) without inclusion of intra-fraction (IF) motion, (c) prefraction transponder positioning with (i) no further correction and corrective action levels (AL) at (ii) 5 and (iii) 3 mm deviations, and (d) prebeam correction with correction strategies (i)–(iii). Analysis for (c): (i) is first conducted using all available motion data measured during each fraction, then again using only motion data when the radiation beam was on. The remainder of the analysis in (c), (ii)-(iii) and (d), (i)–(iii) was performed using only motion data collected while the radiation beam was on. Intratreatment intervention and correction for all excessive motion were simulated as radiation beam gating when a tracking limit was exceeded, followed by position adjustment and immediate resumption of treatment. Repositioning accuracy was simulated using a Gaussian distribution about the planned position with a standard deviation of 0.5 mm as measured with a phantom. 

### 2.2. Removal of Intrafraction Realignments

**Figure 1:**
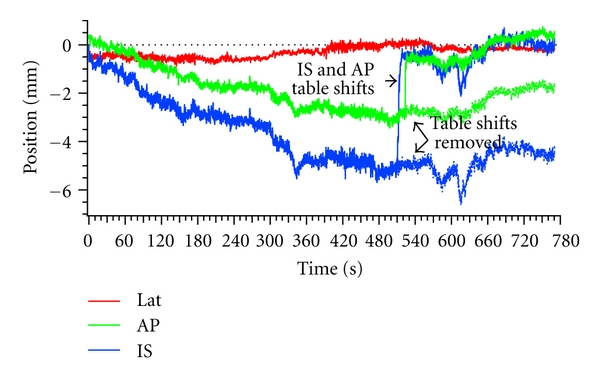
Successive table shifts toward isocenter were present in 8.2% of the measured fraction, such as those seen here in the IS and AP directions. Table shifts were removed to reduce the bias toward isocenter in measured prostate displacements.

As previously discussed, [6] each institution in the study developed its own guidelines on how to adapt treatment when motion occurred during radiation delivery. These ranged from purely observational with no corrective action to stopping the radiation beam and moving the treatment couch to correct persistent deviations from isocenter. Because couch adjustments were used to realign the target volume back to isocenter in 8.2% of all fractions, a bias toward smaller deviations was introduced into the raw data. The recorded realignments were apparent in the raw tracking data as sequential shifts to isocenter in each direction. To reduce this bias for the purposes of this study, these realignments were programmatically removed in a semiautomated manner. The slope and standard deviation of the data prior to the realignment were estimated, and the times at which the realignment were initiated and finished were noted. During the time of the realignment, the trajectory was simulated to have the same slope and standard deviation as that prior to the realignment. The raw data after the realignment was completed were offset to be continuous with the end of the simulated data, as shown in Figure 1.

### 2.3. Margins for Skin-Based Setup with and without Intrafraction Motion

After the target volume is positioned using skin marks, the residual setup error is removed using electromagnetic alignment to the transponders, such that the system reads 0.0 ± 0.5 mm. This residual setup error is used to determine the appropriate PTV margins for setup based on skin marks without inclusion of intra-fraction motion. To include the influence of intra-fraction motion in the margins, these residual setup error values were used to offset the continuously measured intra-fraction motion data to their uncorrected initial positions.

### 2.4. Margins for Pretreatment Setup to Implanted Transponders

In the multi-institutional study, each institution developed their own protocol for use of the system, and in many treatment fractions, comparisons to orthogonal diagnostic X-ray imaging were performed after alignment to the transponders and before the beginning of treatment [6]. Collection of the X-ray images typically took approximately 2–5 minutes, allowing time for the target volume to move from the baseline position. Generally, if deviations were present after X-ray verification, each institution would realign the target volume using the transponders before beginning treatment per the tolerance allowed by their protocol, with treatment beginning 1-2 minutes thereafter. Again, deviations from isocenter due to motion may occur during this slight delay. The mean and standard deviation of initial positions in the left-right (LR), superior-inferior (SI), and anterior-posterior (AP) directions were LR = −0.05 ± 0.43 mm, SI = 0.03 ± 0.80 mm, and AP = −0.11 ± 0.76 mm with ranges of LR = [−3.7,1.2] mm, SI = [−7.9,9.2] mm, and AP = [−4.6,8.3] mm.

In estimating the necessary treatment margins when the target volume is setup to the transponders before each fraction, the preparation and treatment errors due to skin mark setup and inter-fraction motion are assumed to be zero in (1). In addition, intra-fraction motion preparation and treatment errors are first calculated using all the tracking data measured during each treatment fraction, and secondly, including only motion data when the radiation beams were on. 

### 2.5. Margins for Pretreatment Setup to Implanted Transponders with Action Thresholds

Among the protocols implemented during the study, action levels of 3 and 5 mm were established at some institutions. In these cases, the radiation beam was manually gated or delayed until the positional discrepancy was resolved to within tolerance. Some deviations from isocenter were transient excursions that resolved themselves, typically within 20 seconds, while others were drifts that were corrected by couch translations. The frequency of deviations exceeding these action thresholds has previously been reported [6] to be 41% of fractions and 100% of patients for deviations >3 mm, and 15% of fractions and 83% of patients for deviations >5 mm, with large variations among patients and from fraction to fraction for any given patient. 

The corrective actions implemented in this study may take from few minutes to few seconds, depending on the level of automation implemented in the institutions repositioning system. Consequently, when the average position over the last second (10 data points in this case) exceeded an action threshold, the remaining data was simply offset back to isocenter in all directions from that time forward, and the treatment resumed immediately. For calculations in this and following sections, the remote corrections made by the treatment couch had a Gaussian repositioning accuracy of *σ* = 0.5 mm in each direction.

### 2.6. Margins for Prebeam Setup to Implanted Transponders

Because many patients exhibit some drift in the position of the target volume over the course of a single fraction, the preparation and treatment errors over the course of a typical treatment fraction may be significantly larger than that over the course of delivery of a single radiation beam. Consequently, smaller margins are expected if the target volume is repositioned before each radiation beam is initiated. To achieve this frequency of corrective intervention, the couch shifts are made remotely using the machine control interface by the therapists from outside the treatment room so that the next radiation beam is started immediately thereafter. 

Radiation beam on and off times were recorded during the study for the patients considered here. The times were recorded manually by observers and considered to be accurate within about 5 seconds. The preparation and treatment errors for all radiation beams of all patients are added in quadrature to determine population margins for prebeam repositioning.

### 2.7. Margins for Prebeam Setup to Implanted Transponders with Action Thresholds

In these simulations, the target volume was repositioned at the beginning of each radiation beam and corrected back to isocenter if an action threshold was exceeded as previously described. From a calculation perspective, it is again assumed that threshold violation corrections take no time. (No position data was skipped for purposes of this analysis to account for motion that occurred when the radiation beam would have otherwise been off during position corrections.) Again, the preparation and treatment errors during the treatment periods are added in quadrature to estimate appropriate population margins for prebeam setup to implanted markers with action threshold of 3 and 5 mm along each axis.

## 3. Results

**Figure 2:**
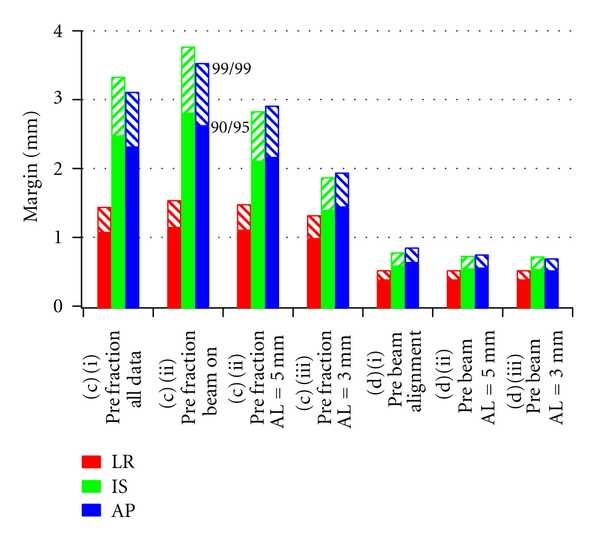
PTV margins for real-time correction strategies for which 90% or 99% of patients receive a minimum dose greater than 95% or 99% of the prescription dose, respectively. Margins based on (c) prefraction, (d) prebeam correction (i) alone, or with further correction at thresholds of (ii) 5 mm and (iii) 3 mm are shown. Margins decrease with increased levels of corrective action.

The results are shown in Figure 2. Positioning by skin marks alone, and ignoring intra-fraction motion, (a), requires margins of (LR,SI,AP)_90,95_ = (13.2,8.2,14.8) mm while inclusion of intra-fraction motion, (b), increases margins to (LR,SI,AP)_90,95_ = (14.7,10.3,15.9) mm. Prefraction positioning, (c), using all data gives margins of (LR, SI, AP) = (1.1,2.5,2.3) mm while correction strategies for (i) no further intervention, (ii) correction to isocenter with a 5 mm action threshold, and (iii) 3 mm action threshold, using only motion data measured when the radiation beam was on, results in margins of (2.4, 4.8, 4.7) mm, (1.1, 2.1, 2.2) mm, and (1.0, 1.4, 1.5) mm, respectively. Prebeam positioning, (d), plus correction strategies (i)–(iii) result in margins of (0.4, 0.6, 0.7) mm, (0.4, 0.6, 0.6) mm, and (0.4, 0.6, 0.5) mm, respectively.

Using values of *α* = 3.36 and *γ* = 0.95 in (1), such that 99% of patients receive a minimum dose of 99% of the prescription dose for strategies (c): (i)–(iii), results in margins of **m**_99,99_ = (LR,SI,AP)_99,99_ = (1.6,3.8,3.6) mm, (1.5, 2.9, 2.9) mm, and (1.4, 1.9, 2.0) mm, respectively. Prebeam positioning, (d), plus correction strategies (i)–(iii) result in margins of **m**_99,99_ = (LR,SI,AP)_99,99_ = (0.6,0.8,0.9) mm, (0.6, 0.8, 0.8) mm, and (0.6, 0.8, 0.7) mm, respectively. These results are shown in Figure 2, with **m**_90,95_ for comparison.

## 4. Discussion

The results presented here were determined from 1157 treatment fractions delivered to 35 patients at 5 institutions. Previous preliminary results were estimated from 11 simulated treatment fractions, each from 11 different patients [5]. Margins for setup to skin marks found here, (LR,SI,AP)_90,95_ = (13.2, 8.2, 14.8) mm, are significantly larger than those reported in the previous work, (LR,SI,AP)_90,95_ = (8.0,10.0,7.3) mm. Additionally, the LR and AP margins are significantly larger than the 10 mm margins commonly used at many institutions. This is largely attributed to the variation among protocols at the 5 institutions which may or may not have placed special emphasis on alignment to skin marks, knowing that the target volume position would subsequently be corrected to isocenter using transponders. Including the intra-fraction data with the skin-based setup increased margins by 1.1 mm to 2.1 mm, which is slightly smaller than the 2-3 mm increase found in the previous study.

In the simplest correction scenario, (c): (i), the target volume is aligned once prior to treatment, similar to the correction strategy used for gold marker implants. Alignment to transponders resulted in margins of (LR, SI, AP) = (1.9,4.1,3.9) mm when all the motion data is used. Because there is a delay of 1-2 minutes before the first, and subsequent radiation beams are turned on, there is time for the prostate to move away from isocenter. Consequently, only using the intra-fraction motion data, when the radiation beam was on, results in margins that are slightly larger, 0.5 mm, in the IS and AP directions. While this is not large, it demonstrates that delays between determining fiducial offsets, making corrections, and beginning treatment lead to larger margins and should be minimized. It should also be noted that these margins are 2-3 mm smaller than previously found from preliminary data [5]. This is attributed to the small number of simulated fractions (eleven) previously available and the relatively large variation that was seen among the eleven subjects.

Strategies that implement action levels of 3 mm or 5 mm, for interventional correction during treatment, (c): (ii)-(iii), allow margins that are 1-2 mm smaller than strategy, (c): (i), where only prefraction alignment was used. This strategy was used at two of the five institutions who participated in this study, with 3 mm and 5 mm action levels, and is used at the University of Michigan with a 3 mm action level. In this strategy, the therapist manually gates the radiation beam when deviations exceed the action level. While this allows for smaller margins, it can increase the time the patient is on the treatment table. It has been reported that, for patients in this study, 77% and 88% of excursions from isocenter resolve themselves within 15 seconds and 30 seconds, respectively [9]. Consequently, a large majority of corrections are resolved in <15–30 seconds without the therapist needing to enter the treatment room which would nominally take 2-3 minutes before resuming treatment. Interfacing of electromagnetic tracking technology with treatment machine control features, such as radiation beam gating and adaptive couch repositioning to correct misalignments between the prostate and radiation beam, will allow these corrections to be made in a more automated fashion and very quickly. It would be possible to make corrections to isocenter before each radiation beam is turned on and when deviations exceed pre-defined action levels. In these cases, scenarios (d): (i)–(iii), the margins to account for intra-fraction motion may be roughly 2 mm or less. It should be noted that the results presented here generally agree with those of Tanyi et al. [10], who found that margins of 1.36 to 2.64 mm were needed with Calypso-based alignment.

In a recent study, Li et al. [11] used the same multi-institutional prostate motion data used here in a dosimetric study to find appropriate PTV margins. In that study, the probability density function (PDF) for each fraction of each of the 35 patients was determined. These were convolved with the static dose distributions of two prostate cases from patients not in the study in order to estimate the dosimetric impact of intra-fraction motion. The correction scenarios simulated in that study were similar to scenarios, (c): (i)–(iii), here, prefraction alignment with no further corrections and corrections based on 3 and 5 mm thresholds. In all three scenarios, they found that a 2 mm margin was adequate to maintain a minimum CTV (prostate gland) dose >95% of the prescription dose in all motion applied to both cases. While this is consistent with the LR population margins found here, it is 0.5 mm to 2 mm smaller than the PTV margins found in the IS and AP directions in this study. It should be reiterated here that the formalism of van Herk, used in this study to determine margins, was also designed to achieve a minimum dose to the CTV of >95% of the prescribed dose, in 90% of patients. As reported by Gordon and Siebers [12], margins found in dosimetric studies, such as those by Li et al. [11], may be smaller than those found with the van Herk margin formula, due to the perfect PTV-to-CTV conformation assumed in the derivation of the formula. While the study by Li et al. used seven uniformly distributed axial radiation beams to achieve a highly conformal IMRT plan, the conformality index of those plans was not reported and must be assumed to be less than perfect.

It should also be noted that prostate rotations were not included here or in the study by Li et al. Other studies have found that the dosimetric impact of prostate rotations is small when 5–10 mm margins are used [13, 14]. However, as margins become smaller, the impact of rotations is expected to become more important. Another study by Rijkhorst et al. showed that when rotations were not corrected in simulation studies, the necessary margin increased from 4 mm to 6 mm to achieve a minimum dose to the CTV >95% of the prescribed dose, in at least 90% of patients [15].

Several years of experience with electromagnetic tracking indicates that the system calibration is quite stable over time, though this has not been quantified. However, a factor that limits the accuracy that may realistically be achieved with the electromagnetic tracking system used in this study is its calibration to isocenter using the room lasers. While lasers are typically checked daily with a 2 mm tolerance, it is likely that they are adjusted to isocenter less frequently, typically monthly, with some small error, perhaps with an accuracy of 0.5 mm. This would lead to a small systematic error in tracking system calibration that would likely change direction and magnitude, once or twice during a prostate patient treatment, which typically lasts 8 weeks. Because margins are sensitive to systematic errors, whose standard deviations are added in quadrature, small errors in system calibration will become more important for margins below a few millimeters. Obviously, great care should be taken to calibrate the lasers as accurately as possible before calibrating the tracking system.

Consequently, the margins calculated here only represent those which are needed to account for intrafractional translational motion using the various intervention scenarios studied. The sub-millimeter results, shown in Figure 2, suggest that as electromagnetic guidance becomes more integrated loss of dosimetric coverage due to translations will become very small compared to other uncertainties. In particular, the accuracy of the system relative to radiation isocenter must be studied in greater detail, and the impact of deformations and rotations on dosimetric coverage must be further investigated. Due to other uncertainties mentioned above, margins below 5 mm should only be used with great care to monitor and minimize their effects.

Finally, electromagnetic tracking offers the opportunity to pursue greater consistency in outcomes by seeking to deliver a minimum dose to the CTV, which is a higher percentage of the prescription dose, to a higher percentage of patients. Because the systematic and random errors in determining the position of the target volume can be greatly reduced through increasing levels of real-time correction, it becomes possible to choose parameters for the margin recipe such that 99% of patients receive a minimum dose of 99% of the prescription dose, while using margins that are smaller than the 5 to 10 mm margins commonly used today.

## 5. Conclusions

The ability to continuously monitor intra-fraction prostate motion has a significant impact on the population margins required for prostate treatment. Increasing degrees of corrective intervention have the potential to reduce PTV margins to approximately 2 mm while simultaneously allowing a larger percentage of patients (99% versus 90%) to receive a minimum dose which is a higher percentage (99% versus 95%) of the prescription dose. Reduced motion and margins result in less dose to healthy surrounding tissue and higher minimum dose to the intended target, which may lead to fewer complications and improved control rates.

##  Conflict of Interests

James M. Balter is a scientific consultant to Calypso Medical Technologies, Inc. Lisa Levine is an employee of Calypso Medical Technologies, Inc. Both have a financial interest in Calypso Medical Technologies, Inc.
